# Effect of ischemic postconditioning on myocardial protection in patients undergoing coronary artery bypass grafting surgery with cardiopulmonary bypass

**DOI:** 10.15171/jcvtr.2016.13

**Published:** 2016-06-28

**Authors:** Nasser Safaei, Mohammad Ali Sheikhalizadeh, Reza Badalzadeh

**Affiliations:** ^1^Cardiovascular Research Center, Tabriz University of Medical Sciences, Tabriz, Iran; ^2^Department of Physiology, Faculty of Medicine, Tabriz University of Medical Sciences, Tabriz, Iran

**Keywords:** Ischemic Postconditioning, Coronary Artery Bypass Grafting, Reperfusion Injury

## Abstract

***Introduction:*** Reperfusion injury is a well-known phenomenon following restoration of the coronary circulation after coronary artery bypass grafting (CABG) that impairs myocardial function. In order to control the severity of this injury, we aimed to investigate the effect of a new conditioning strategy namely ischemic postconditioning (IPOC) along with controlled aortic root reperfusion (CARR) on myocardial protection in CABG surgery with cardiopulmonary bypass.

***Methods:*** In a doubled blind clinical trial study, 51 patients undergoing first-time elective CABG were randomly divided in three groups: CARR, IPOC, and combination of IPOC and CARR. At the end of procedure and just before aortic cross-clamp removal, reperfusion was started as following: In CARR-receiving groups, the reperfusion was started with low perfusion pressures for 10 minutes, and in IPOC-receiving groups, three cycles of 1 minute episodes of ischemia separated by 1 minute episodes of reperfusion was applied as postconditioning protocol. Left ventricular ejection fraction (EF) (by echocardiography), inotrope requirement index, and myocardial arrhythmias were measured up to 72 hours after operation.

***Results:*** Echocardiography revealed that the recovery of EF after operation in IPOC group was significantly higher than those of two other groups (P < 0.05). Inotropic support requirement was significantly lower in IPOC groups. In addition, the incidence of atrial and ventricular arrhythmias after opening of aortic clamp and in intensive care unit (ICU) as well as recovery time of cardiac rhythm upon reperfusion were lowered by administration of IPOC, as compared with CARR group.

***Conclusion:*** The study suggests that IPOC may provide clinical benefits against reperfusion injury in patients undergoing CABG surgery and maintain the post ischemic left ventricular performance.

## Introduction

In patients undergoing open heart surgery with the cardiopulmonary bypass, the coronary blood flow and myocardial demand is not normal and thus the myocardial ischemia/reperfusion (I/R) injury is susceptible, often due to the various reasons such as aortic clamp, injection of cardioplegic solution, heating and cooling procedures etc,^1^ In the first seconds of ischemia, the aerobic metabolism becomes anaerobic. It has been long known that ischemic injuries deplete the cell energy, resulting in dysfunction of cellular transporters and thereby increased intracellular calcium concentration that ultimately causes damages to the cells in the form of inflammation or necrosis.^1,2^ These processes may lead to the myocardial diastolic and systolic dysfunctions. Myocardial I/R injury can be evaluated in three types including muscular, conductive, and endothelial manifestations. Restoration of perfusion to the ischemic tissue, in spite of its necessity, may cause further electrical and mechanical complications, which they are more severe than the ischemic injuries.^3^

Ischemic postconditioning (IPOC), introduced by Zhao et al in 2003, has been demonstrated as a reliable cardioprotective technique in animal I/R experiments.^4^ In this protocol, very short and alternative episodes of reperfusion and ischemia are applied immediately at the onset of reperfusion, and this is capable to improve ventricular dysfunction and reduce infarct size by approximately 50%.^5,6^ It has been suggested that the underlying mechanisms of cardioprotection by IPOC are diverse including the inhibiting inflammatory response and formation and release of autacoids and cytokines, maintaining acidosis during early reperfusion, maintaining mitochondrial function and reducing the opening of its permeability pores, activating signaling protein kinases, increasing nitric oxide release, increasing adenosine receptor stimulation and several other mechanisms.^6-10^

The cardioprotective effects of IPOC phenomenon has been reported in the setting of percutaneous coronary intervention for acute myocardial infarction (MI),^11^ valve replacement^12^ and tetralogy of Fallot.^13^ The results of these studies on IPOC in humans as well as in experimental animals are very encouraging. However, whether IPOC exerts protection in coronary artery bypass grafting (CABG) surgery with cardiopulmonary bypass and whether it has an additive effect to controlled aortic root reperfusion (CARR) protocol in the setting of cardiac surgery remains to be elucidated. Therefore, due to the imposing of various degrees of ischemia and reperfusion injuries to the hearts of patients undergoing CABG surgery, and in order to the reduction of the resultant I/R injuries, the aim of this study was to investigate the effect of IPOC on myocardial protection in CABG surgery with cardiopulmonary bypass and comparing its effect with those of routinely-used CARR protocol.

## Materials and Methods

### Patients

In this prospective, cross-sectional study, 51 cardiac patients undergoing elective CABG referred to Shahid Madani hospital (Tabriz, Iran) were enrolled to the study according to the inclusion criteria. The inclusion criteria included as age 50-70 years old, no previous history of heart surgery, complete disruption of anterior descending coronary artery, lack of severe left ventricular dysfunction; no MI with Q wave in past six weeks, no emergency surgery, and no hemodynamic instability with inotropic support before surgery. In addition, during the surgical operation, the patients faced with extra interventions such as valve surgery and endarterectomy on complicated vessels, or with clamp time more than 100 minutes and pump time more than 130 minutes were excluded from the study.

### Sample size and grouping

The sample size of the study was calculated using related software (PS software for Power and Sample Size Calculations), with considering the significance level set at 0.05 and power of study set as higher than 80%, based on the results of previous studies. The resultant sample size was 15 patients for each group; however, for increasing the accuracy of this experiment, the sample size per group was increased to 17 patients. The eligible patients for the study were selected online using the website http://www.graphpad.com/quickcalcs/ and randomly divided into three groups, including CARR group which considered as control, IPOC group and combination (CARR plus IPOC) group.

### Operation protocol

**Figure 1:**
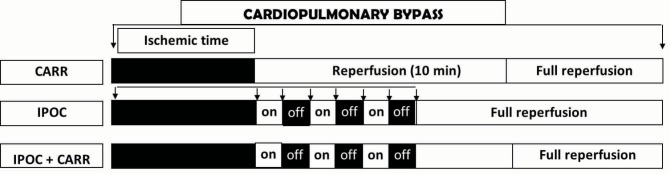
Experimental protocol in three groups.

All patients who had provided written consent were underwent surgery with the help of cardiopulmonary bypass. The membranous oxygenator was used to calculate the non-pulsatile blood flow. The patient’s temperature (nasopharyngeal) was reached to about 32°C using a heater-cooler system. After the cross-clamp of aorta, the cold blood cardioplegic solution with the formula of St. Thomas (Martindale Inc.) and the amount of 150 cc/m^2^/min for 4 minutes was antegradely administered through the cannula placed in the aortic root. The half of the initial dose was repeated every 20 minutes and the concentrations of potassium in solution were kept at 16 meq/l and 10 meq/l, respectively. After the end of CABG, the patients were warmed up and the coronary flow to their hearts was started with opening of the aortic clamp through the aortic root in three groups, as following: (*a*) in CARR group; in early 10 minutes of reperfusion, coronary reperfusion with the pressure of 30 mm Hg for initial 3 minutes and with the pressure of 50 mm Hg for the next 7 minutes; (*b*) in IPOC group; in early 6 minutes of reperfusion with systemic pressure, three cycles of intermittent 60 seconds reperfusion and 60 seconds ischemia was applied using bulldogs placed on the vascular grafts; and (*c*) in combination group; in early 6 minutes of reperfusion with the pressure of 30 mm Hg, three cycles of IPOC was applied as above and then the reperfusion with a pressure of 50 mm Hg was continued for the next 4 minutes. In all groups, after management of initial 10 minutes of reperfusion, the reperfusion was allowed to be continued with the normal systemic perfusion pressure of the coronary artery (Figure 1). All patients were then subjected to measurements of ejection fractions (EFs), arrhythmias and inotropic requirements for 3 successive days in intensive care unit (ICU) and cardiac ward.

### Measurement of ejection fraction of the hearts

Echocardiography was used to evaluate the surgical result after the operation and left ventricle ejection fraction (EF). Echocardiographic imaging was recorded before surgery and fifth day after operation. The left ventricle performance was visualized in apical, 4-chamber and long-axis views. The cardiologist who measured EF before and after the procedure was blinded to patient’s grouping.

### Intraoperative variables

The number of coronary bypass grafts, duration of cardiopulmonary bypass, and duration of aortic cross-clamp, pump time, mechanical ventilation time, and requirement for inotropic drugs were evaluated during the operation time. After surgery, all patients were admitted to the ICU and received the similar postoperative care as determined by the caring physicians.

### Endpoints

The endpoints after operation were recovery time of cardiac rhythm at the first minutes of reperfusion, myocardial arrhythmias after opening of aortic clamp, myocardial arrhythmias in ICU, needs to D/C shock, length of ICU stay, postoperative 3 day mortality, prolonged ventilation time, as well as postoperative inotropic requirement, and left ventricular EF. In addition, the inotropic score for the first 72 hours post-operation time was calculated with the formula: [dobutamine × 1] + [(epinephrine + norepinephrine)] × 100. The inotropic support was given on the basis of hemodynamic and clinical conditions and doses of drugs was titrated according to the hemodynamic parameter. If needed, dobutamine, norepinephrine or epinephrine were added for sufficient hemodynamic support.

### Statistical analysis

All values were expressed as mean ± standard error of the means. The data of parametric variables between three groups were analyzed using one-way Analysis of variance (ANOVA) followed by Tukey post hoc test. Differences were considered statistically significant when *P *< 0.05.

## Results

### General characteristics of patients

General characteristics of patients in three groups have been shown in Table 1. The mean time of surgery in CARR group was 5.05 ± 07 hours, in IPOC group was 5.2 ± 0.75 hours and in CARR+IPOC group was 4.9 ± 0.53 hours which these times were not significantly different from each other. In addition, the time for using the cardiopulmonary pump among groups were measured and the results showed no significant changes between groups. The cardiopulmonary pump times were as for CARR group 91.52 ± 21.65 minutes, for IPOC group 104.2 ± 32.99 minutes and for combination group 100 ± 22.8 minutes. The durations of aortic cross-clamp for three groups were 51.29 ± 13.35, 56.47 ± 14.16, and 53.29±9.99 minutes, respectively. Again, there was no statistically significant difference in duration of aortic cross-clamp (Table 1).

**Table 1:** General characteristics of patients in three groups

**Variables **	**Groups**		
	**CARR**	**IPOC**	**CARR+ IPOC**
Gender (F/M)	8/9	7/10	8/9
Age (years old); Mean‏±SD	60.3±6.89	58.1±6.96	58.6±6.17
Body weight (kg); Mean‏±SD	73.1±10.81	78±11.71	74±11.16
BMI; Mean‏±SD	26.54±2.93	27.44±2.80	28.05±3.96
Preoperative risk factors			
Smoking; n (%)	5 (6.41)	5 (6.41)	6 (7.69)
Hyperlipidemia; n (%)	3 (3.85)	4 (5.13)	8 (10.26)
Hypertension; n (%)	11 (10.40)	9 (11.54)	10 (6.41)
Controlled diabetes; n (%)	5 (6.41)	7 (8.97)	5 (6.41)
Total operation time (h); Mean‏±SD	5±0.7	5.2±0.75	4.9±0.53
Pump time (min); Mean‏±SD	91.5±21.65	104.2±32.99	100±22.80
Aortic cross-clamp time (min); Mean‏±SD	51.29±13.35	56.47±14.16	53.29±9.99
Mechanical ventilation time (h); Mean‏±SD	16.6±11.25	16.9±18.47	15.9±10.71
Number of grafted vessels (n); Mean‏±SD	2.90±0.74	3±0.70	2.9‏±0.68

Abbreviations: CARR, controlled aortic root reperfusion; IPOC, ischemic postconditioning; F, female; M, male; SD, standard deviation; n, number; BMI, body mass index.

### Recovery time of the heart rhythms

On the other hand, the recovery time for the heart rhythms after opening of the clamp were analyzed between groups and the results indicated that this rhythm recovery time in CARR group was 6.25 ± 3.45 minutes, in IPOC group was 4.58 ± 1.62 minutes and in CARR+IPOC group was 5.52 ± 2.47 minutes (Table 2). The statistical analysis showed that IPOC lowered the recovery time about more than one minute as compared with CARR group; although this effect was not statistically significant, but it may have clinical importance.

**Table 2:** Cardiac rhythm recovery and arrhythmias in patients of three groups

**Variables**	**Groups**		
	**CARR**	**IPOC**	**CARR+ IPOC**
Time of cardiac rhythm recovery (min); Mean‏±SD	6.29±3.45	4.58±1.62	5.52±2.47
D/C shock requirement after aortic-clamp opening; n (f)	6 (11)	2 (3)	3 (6)
Myocardial arrhythmias after aortic-clamp opening; n (type)	6 (VF)	2 (VF)	2 (VT) +3 (VF)
Myocardial arrhythmias in ICU; n (type)	4 (AF)+ 1 (PVC)	1 (PAC)	-

Abbreviations: CARR, controlled aortic root reperfusion; IPOC, ischemic postconditioning; SD, standard deviation; n, number; f, frequency; PVC, premature ventricular complex; AF, atrial fibrillation; VT, ventricular tachycardia; VF, ventricular fibrillation.

### Electrical (D/C) shock requirement during cessation of the cardiopulmonary bypass

Six patients in CARR group, totally 11 times (54.4%), 2 patients in IPOC group, totally 3 times (18.18%), and 3 patients in CARR+IPOC group, totally 6 times (27.27%) received electrical D/C shock due to the arrhythmias after the opening of the aortic cross-clamp (Table 2). The results showed that the electrical shock requirement (as an indicator for the myocardial protection evaluation during cardiac ischemia) in IPOC group was the lowest as compared with other groups, while 35% of patients of CARR group received the electrical shock mandatorily because of lethal arrhythmias that have not relieved by medicinal treatments.

### The frequency of myocardial arrhythmias after opening the aortic clamp

After opening the aortic cross-clamp, no types of atrial arrhythmias were observed in patients of any groups. However, the ventricular arrhythmias were detected in 6 patients of CARR group as ventricular fibrillation (VF), in 2 patient of IPOC group as VF, and in 5 patients of CARR+IPOC group as VF and ventricular tachycardia (VT) (Table 2).

### The frequency of myocardial arrhythmias in post-operation days

In ICU for 72 hours, all patients were carefully monitored for the incidence of cardiac arrhythmias. According to the findings of this monitoring, most arrhythmias were occurred in CARR group so that, 4 atrial arrhythmias as atrial fibrillation (AF) and one ventricular arrhythmia as premature ventricular complex (PVC) were observed in patients of CARR group. On the other hand, in IPOC group only one patient showed a premature atrial contraction (PAC) which is the least harmful cardiac arrhythmia. In addition, no cardiac arrhythmia was observed in the combination group (Table 2).

### The inotropic requirement of patients

Scoring of the inotropes requirement in terms of type and dose of inotrope drugs was performed based on the recent articles and in conjunction with anesthesiologists. In operation room, five patients of CARR group with a score of 7.20 ± 4.28 required the inotropes and this was significantly higher than those of IPOC group (4 patients with a score of 4.41 ± 4.99) and CARR+IPOC group (5 patients with a score of 4.11 ± 12.02) (Table 3). Similarly in ICU, which the received amounts of inotropes were monitored for 72 hours, six patients from CARR group (with a score of 11.76 ± 6.55), one patient from IPOC group (with a score of 1.76 ± 1.70), and 4 patients from CARR+IPOC group (with a score of 3.82 ± 2.92) received inotrope drugs (Table 3). These results indicated that patients in IPOC group needed lower amount of inotrope support in comparison with the other groups.

**Table 3:** The inotropic requirement of patients in three groups

**Variables**	**Groups**		
	**CARR**	**IPOC**	**CARR+ IPOC**
Inotropes received in operation room (n/score)	5 (7.20±4.28)	4 (4.41±4.99)	5 (4.11±12.02)
Inotropes received in ICU (n/score)	6 (11.76±6.55)	1 (1.76±1.76)	4 (3.82±2.92)

Abbreviations: CARR, controlled aortic root reperfusion; IPOC, ischemic postconditioning; ICU, intensive care unit.

### Changes in left ventricular ejection fraction in patients

**Figure 2:**
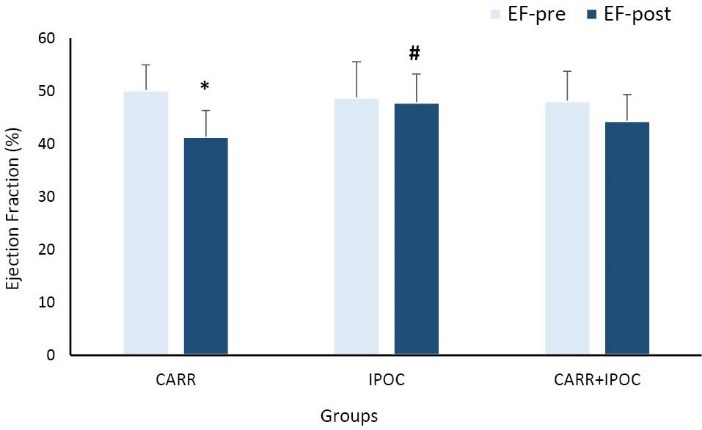
The alterations of left ventricular ejection fraction (EF, %) in the preoperative and postoperative periods in patients of three groups. *P<0.05 as compared with those of EF-pre of CARR group; ^#^P<0.05 as compared with those of EF-post of CARR group. EF-pre: preoperative ejection fraction, and EF-post: postoperative ejection fraction.

**Figure 3:**
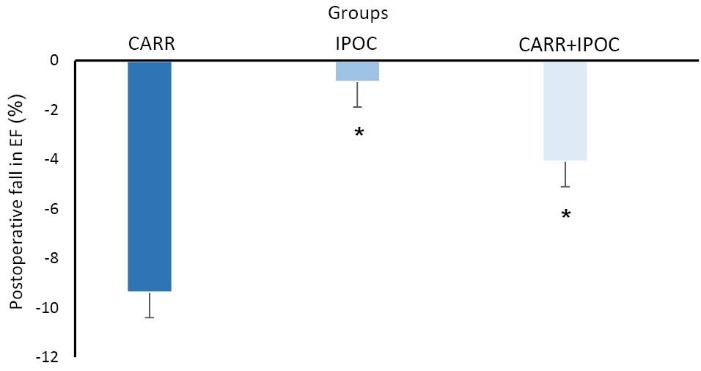
The differences in fall of left ventricular ejection fraction (EF, %) after operation in patients of three groups. *P < 0.05 as compared with CARR group.

The left ventricular EF was measured in the preoperative and postoperative periods as one of the most important indicators in the evaluation of myocardial protection. The preoperative left ventricular EF in CARR group was 50.9 ± 4.75%, in IPOC group was 48.8 ± 6.73% and in CARR+IPOC group was 48.2 ± 5.57% which there was no significant difference between groups (Figure 2). However, in fifth day after operation, the left ventricular EF in patients of CARR group was 41.47 ± 4.9%, while in IPOC group it was 47.49 ± 5.32% and in CARR+IPOC group was 44.41 ± 4.96%. The results indicated that the recovery of EF after operation in IPOC group was significantly higher than those of two other groups (*P *< 0.05), so that the postoperative EF was reduced only about 0.88% in IPOC group in comparison with its preoperative value. This reduction for CARR group was about 9.4% and for combination group about 4.11% (Figure 2 and Figure 3).

## Discussion

In the present study, we evaluated the effect of IOPC in reducing the ischemia-reperfusion injury (I/R) immediately at the opening of aortic cross-clamp in patients undergoing coronary artery bypass graft surgery by cardiopulmonary bypass and compared its effects with those of CARR method.

The basic idea for reduction of myocardial reperfusion injury was introduced first by Buckberg and colleagues in 1970^14^; they used a protocol similar to our CARR in animal studies which its aim was to control the velocity of blood flow in the first few minutes of reperfusion to the ischemic area. The outcomes of their protocol were reduced cell edema and improvement of myocardial performance. In mid-1980, Braunwald and Kloner again raised the issue of reperfusion injury.^15^ Thereafter, a variety of medicinal compounds were used, for about 20 years in experimentations, to overcome this injury, including beta-blockers, anti-inflammatory drugs, free radicals scavengers, chelating agents such as calcium citrate-phosphate- dextrose, sodium-hydrogen ion exchange inhibitors, calcium channel blockers and adenosine, all of which were associated with good results. However, the inability to transfer this experience in clinical settings, side effects of medications as well as persistence of reperfusion injury despite the application of the mentioned compounds led to the introduction and application of “conditioning” strategies, especially after ischemic insult, such as IPOC.^10^

There are no enough clinical studies on the cardioprotective effects of IPOC, and reaching to a precise conclusion about its effectiveness in few studies is difficult due to different endpoints investigated and diverse results reported. In the present study, we showed that the IPOC significantly improved the inotrope requirement score as well as left ventricular EF. Evaluating the type of administered inotrope drug and amount of postoperative inotrope support in operation room and in ICU showed that IPOC group have received significantly the lowest amounts of inotrope between groups; only 29% of patients in IPOC group vs. 64% and 52% in CARR and combination groups, respectively. These findings were in accord with the results of Wanjun et al^12^ in adult heart valve replacement, Li et al^13^ in corrections of tetralogy of Fallot, and Zhen-Xiao et al^16^ in heart valve replacement surgery, via pharmacological postconditioning with adenosine. However, Durdu et al^17^ could not find a statistically significant reduction in the need for inotrope support in patients, by a modified mechanical postconditioning.

One of the most important indices of myocardial function is the left ventricular ejection fraction or EF. Undoubtedly, the severity of myocardial injury is directly associated with the reduction in EF. The findings of this study indicated that myocardial injury in patients of IPOC group was lesser than the other groups. The measurement of left ventricular EF before and after surgery showed only 0.88% reduction in EF of IPOC patients that is comparably lower than 9.4% reduction of EF in CARR group. In other words, IPOC strategy could significantly prevent the fall of EF after surgery and considerably improved the EF by about 9% in comparison with the CARR control group. Similar results have been reported about the effect of IPOC on the other surgeries.^12,18,19^ In patients undergoing angioplasty, EF in IPOC group was 23% higher than those of control group.^19^ Some studies also showed statistically significant decrease in EF and better results by IPOC protocol only after a relatively long period (one month and one year after surgery) but not in the early periods.^7,20^ In another study, IPOC also decreased the myocardial infarct size and area at risk, in addition to the improvement of left ventricular EF.^18^ Altogether, these findings indicated the cytoprotective influences of IPOC in myocardial ischemic insults.

The incidences of atrial and ventricular arrhythmias are considered as indications of damage to the cardiac conductive cells during restoring blood flow to the ischemic heart. After releasing the aortic clamp, we observed the ventricular arrhythmia only in one patient of IPOC group as compared to significantly higher incidence of arrhythmias in the other groups, indicating the antiarrhythmic effects of IPOC in this study. In addition, the number and frequency of electrical shock administration to relief the drug-resistant arrhythmias were significantly lower in IPOC patients. More importantly, the recovery time to return the heart rhythm after opening the aortic cross-clamp was lower in IPOC than other groups (although not statistically significant). This recovery rhythm could be used as a clinical indicator for determining the severity of reperfusion injury. Although as a limitation in the present study, we could not take into account the underlying mechanisms of the effects of IPOC on clinical manifestations, its protective influences can be attributed to its multiplex actions, based on previous reports. Along with improving the blood supply to the nodal and conductive cells of the heart, IPOC also may directly influence the function of these cells. Moreover, IPOC may impact on endothelial cell layer to improve their integrity and function in releasing cytoprotective agents and reduce the risk of adhesions of neutrophil cells to the endothelial layer and induction of inflammatory and oxidative reactions.^4^ The other mechanisms would play important roles in the cardioprotective effects of IPOC include the activation of cell signaling protein kinases such as PKC, PI3K/Akt, and eNOS or the opening of mitochondrial ATP-sensitive potassium (mitoK-ATP) channels and finally the inhibition of mitochondrial permeability transition pore (mPTP).^21-23^ The mPTP and mitoK-ATP channels play important roles in the regulation of mitochondrial membrane potential and matrix size and volume, by which they preserve the mitochondrial function and integrity during I/R injuries and thus protect the heart against these circumstances.^9,24-26^

In conclusion, our results indicated that IPOC in the setting of I/R injuries induced by CABG with CPB in patients could improve the left ventricular EF after surgery, reduce the level of inotrope requirements and exert antiarrhythmic influences. These findings support the cardioprotective effects of IPOC in these patients, similar to or even better than those of CARR effects in most endpoints, in spite of lower sample size in this study. Moreover, we reported the short-term effects of the protocols, but following-up patients for assessing the consistency of IPOC effects would provide more reliable comparison. Therefore, further clinical trials with larger population size are warranted to explore the long-term outcomes of IPOC as well as exact underlying molecular mechanisms of this therapeutic strategy in I/R conditions of human heart.

## Ethical Approval

The study protocol was approved by the ethics committee of Tabriz university of medical sciences (no: 93109; 09/10/2014; IRCT2014121520324N1). Written consents were obtained from all patients prior to inclusion in the study.

## Competing of interests

The authors have no conflicts of interest in regard to this research or its funding.

## Acknowledgements

This study was derived from the MSc thesis of MA Sheikhalizadeh (NO: 92.2-5.8) and supported by a grant from Cardiovascular Research Center, Tabriz University of Medical Sciences.
